# Supplementary material for: Predicting visual function by interpreting a neuronal wiring diagram
Source: Nature. 2024 Oct 2;634(8032):113–23. doi: 10.1038/s41586-024-07953-5 (PMC11446822; doi:10.1038/s41586-024-07953-5)
Supplement: Supplementary file 6 — CRF and ERF predictions for individual TmY4 and TmY9 cells. Analogous to Supplementary Data 3, but for TmY target types. Shown are the top four monosynaptic pathways, the strongest pathway passing through each of the top ten intermediary types (ranking from Extended Data Fig. 7), and the trisynaptic pathway Tm1–TmY–Dm3–TmY (see the section entitled Prediction of spatial normalization). [file 41586_2024_7953_MOESM6_ESM.zip › DataS4/TmY9q/720575940625062989.pdf]

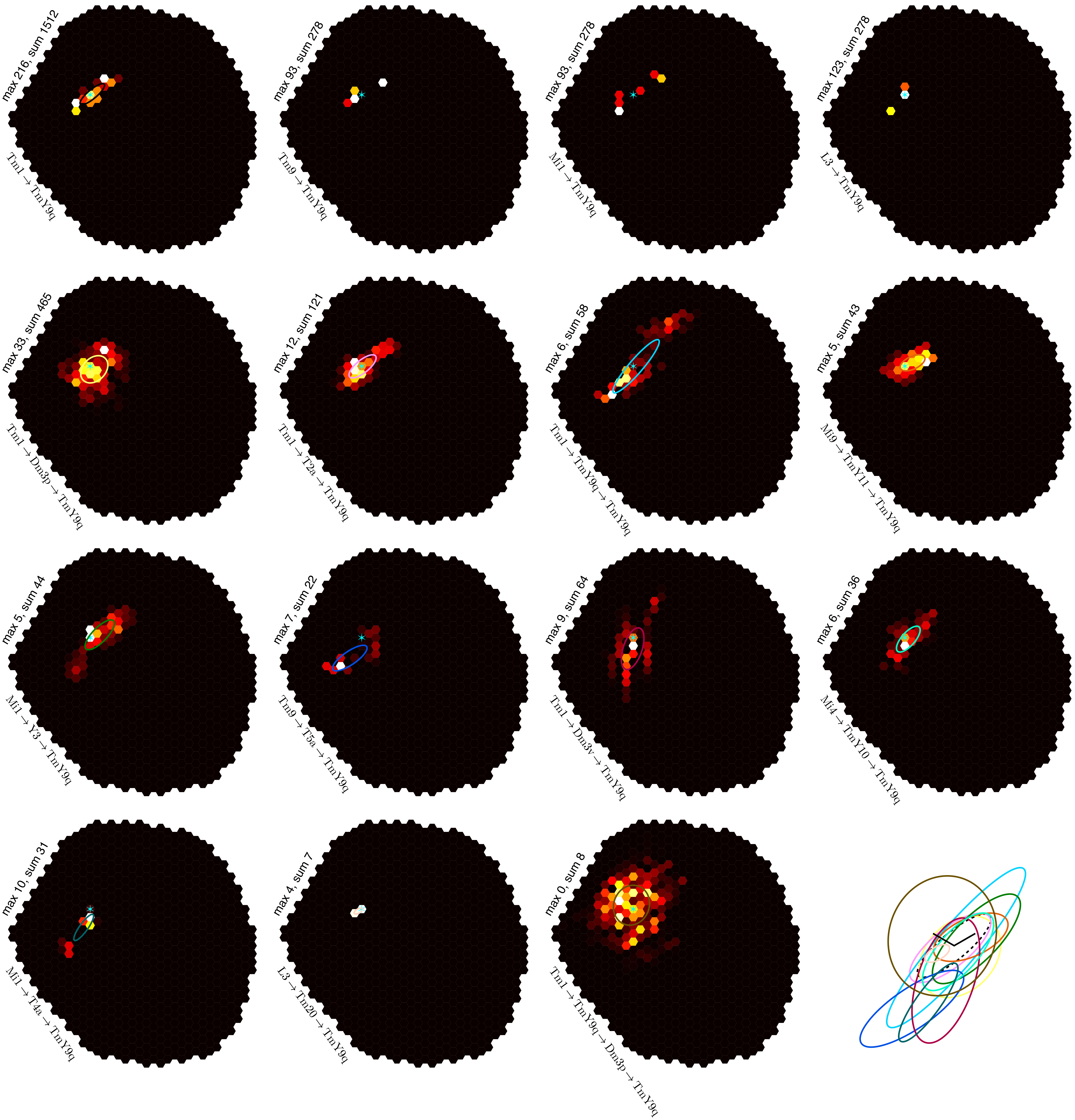
max 216, sum 1512
Tm1 → TmY9q
max 93, sum 278
Tm9 → TmY9q
max 93, sum 278
Mi1 → TmY9q
max 123, sum 278
L3 → TmY9q
max 33, sum 465
Tm1 → Dm3p → TmY9q
max 12, sum 121
Tm1 → T2a → TmY9q
max 6, sum 58
Tm1 → TmY9q → TmY9q
max 5, sum 43
Mi9 → TmY11 → TmY9q
max 5, sum 44
Mi1 → Y3 → TmY9q
max 7, sum 22
Tm9 → T5a → TmY9q
max 9, sum 64
Tm1 → Dm3v → TmY9q
max 6, sum 36
Mi4 → TmY10 → TmY9q
max 10, sum 31
Mi1 → T4a → TmY9q
max 4, sum 7
L3 → Tm20 → TmY9q
max 0, sum 8
Tm1 → TmY9q → Dm3p → TmY9q
